# Study on the impact of central environmental protection inspection on the health of the older adult population—A quasi-natural experiment in China

**DOI:** 10.3389/fpubh.2024.1290192

**Published:** 2024-01-23

**Authors:** Jiayu Yang, Juqiu Deng, Lianguang Ye, Li Liu, Xiuying Hu

**Affiliations:** ^1^West China Hospital of Sichuan University/Innovation Center of Nursing Research/Sichuan Key Laboratory of Nursing, Chengdu, Sichuan, China; ^2^School of Economics, Sichuan University, Chengdu, Sichuan, China

**Keywords:** central environmental protection inspection, health of the older adult population, local governments, environmental governance, quasi-natural experiment

## Abstract

In 2015, the central government of China established the Central Environmental Protection Inspection (CEPI) system for oversight of local governments. It enhanced local government enforcement of environmental regulations, which had a considerable influence on the health of the local older adult population. This study quantifies the effects of local government regulation brought about by CEPI on the health of the older adult. It examines the impact mechanism using the DID model and panel data from the China Health and Retirement Longitudinal Study (CHARLS). The results show that (1) local governments’ environmental protection regulations implemented by CEPI have a positive impact on the general health of the older adult. The results of the study passed the parallel trend test, PSM test, replacement variable test, and placebo test and remained significant; (2) in terms of the impact mechanism, CEPI has promoted local governments’ environmental governance initiatives, which has reduced industrial wastewater emissions, industrial sulfur dioxide emissions, and industrial fumes emissions. This has improved air quality, thereby creating a good living environment for the older adult and improving their overall health; (3) according to heterogeneity research, the health of older adult living in the Yangtze River Basin, urban older adult, and older adult without chronic diseases is more significantly affected by the environmental protection regulations of the local governments brought about by CEPI.

## 1 Introduction

In the face of a rapidly aging global population, nations worldwide are confronted with the multifaceted challenge of ensuring the wellbeing of their older adult citizens. This demographic shift is particularly pronounced in China, where the aging population has reached unprecedented levels. As per the United Nations’ “World Population Prospects 2022” report, the proportion of the global population aged 65 and above is expected to increase from 10 to 16% between 2022 and 2050 (1). Notably, in 2021, the population of people aged 65 and above in China reached 200 million, comprising 14.2% of its total population (2). As the world’s most populous nation with a burgeoning older adult demographic, China’s proactive response to the issue of population aging holds immense significance, not only within its national context but also for global socioeconomic sustainability.

The World Health Organization in its “World Report on Ageing and Health” in 2016 defined “healthy aging” as the process of developing and maintaining the functional ability essential for wellbeing in old age. This functional ability denotes the health-related factors that allow individuals to live and act in alignment with their personal preferences and beliefs. It encompasses an individual’s intrinsic capacity, relevant environmental characteristics, and the interaction between the two. Intrinsic capacity refers to the combined physical and cognitive abilities that an individual can harness. Environmental characteristics comprise all external factors that shape an individual’s living context (3). This suggests that the environment plays an important role in fostering healthy aging. Alarmingly, the World Health Organization’s reports indicate that millions of deaths annually can be attributed to air and water pollution. Based on the findings of the World Health Organization’s survey in 2018, over seven million people worldwide die each year due to air pollution, accounting for approximately 25% of global mortality (4). The 2019 Global Air Quality Report reveals that nearly five million people worldwide have succumbed to death due to prolonged exposure to air pollution. Additionally, according to the World Health Organization’s report in 2023, 80% of diseases worldwide are associated with water pollution (5). Hence, it is evident that environmental pollution has become one of the significant factors endangering human health. Notably, environmental pollution exerts a more substantial adverse influence on the health of the older adult compared to healthier individuals and younger generations (6). In this regard, the older adult are more vulnerable to the detrimental consequences of air pollution (7). Meanwhile, high environmental pollution levels, as evidenced by research (8), pose a serious threat to the health and longevity of the older adult. Numerous studies have explored the salient link between environmental pollution and the health of older individuals (9, 10). Nevertheless, few studies have concentrated on the impact of CEPI on the health of the older adult.

Given the crucial role of environmental governance in the impact of environmental pollution on public health, in July 2015, China implemented the “Environmental Protection Inspection Program (for Trial Implementation),” marking the first instance of central government-led oversight of local governments’ environmental protection performance.^1^ The program dispatched inspection teams to various localities in batches to conduct on-site evaluations of the environmental governance of local governments and to handle local complaints and cases. The first round of CEPI was piloted in Hebei and quickly covered the whole country, during which more than 18,000 local cadres were held accountable (11). It has significantly reduced industrial chemical oxygen demand (COD) emissions (12) and has promoted long-term improvement in environmental performance (13).

This study attempted to address the following questions: First, is there a correlation between the implementation of CEPI and the improvement of the health level of the older adult population in the same period? That is, has CEPI improved the overall health of the older adult? Second, what is the impact mechanism of the correlation? Is it related to the substantial increase in local governments’ incentives for environmental governance? Specifically, in which areas of governance and for which particular pollutants could reductions in their emissions lead to significant improvements in the health of the older adult population? Third, given the vastness of China and the enormous variations in regional development, is the impact of CEPI on the health of the older adult population universal? Will it fluctuate due to factors such as location-specific and urban–rural dichotomy? To answer these questions, the study selected a self-assessed health score (*Shealth*) to measure the health status of the older adult population. The analysis was based on the panel data of the China Health and Retirement Longitudinal Study (CHARLS) from 2011 to 2018. This study adopted the multi-temporal double-difference-in-differences (DID) model to investigate the impact of CEPI on the health of the older adult population, as well as to explore the underlying mechanisms of impact and the display of heterogeneity.

## 2 Literature review

Two research areas are closely related to this study. One focuses on whether specific policy measures such as CEPI have a long-lasting and steady positive influence on the environment. Another concern is the impact of the environment on the health of the older adult.

CEPI is a unique environmental regulatory policy. A large number of scholars have regarded it as a campaign-style enforcement system and examined the outcome of its implementation. Most of the research results show that CEPI achieved immediate results in environmental governance in the short term (13–17). In addition, Jia and Chen argued that the experience accumulated from the previous inspections greatly contributed to the subsequent performance of CEPI, making this inspection system more effective in the long run. The impact of the CEPI reportedly appeared to be long-lasting and optimal even after the CEPI (13). This effect is attributed to the credible central commitment and effective external incentives provided by the CEPI system at the central government level, which disrupts the existing incentive structure of local governments. By reprioritizing environmental protection, it integrates all sectors and all pollutants into a unified management framework (18). The cyclical inspections, on the other hand, have served as a wake-up call for local governments that the old way of development (that is, pursuing economic growth at the expense of the ecological environment) will no longer work, leading them to put a greater emphasis on environmental governance (19). However, the CEPI system requires additional improvement in terms of environmental co-responsibility execution, and some policies require legislative reinforcement (16). In terms of social participation, the CEPI welcomes and encourages citizens to file pollution-related complaints, which not only promotes the awakening of the public’s environmental awareness but also promotes the public’s participation in environmental protection (20). In addition, the air quality conditions and changing trends of the cities with inspection and their neighboring cities without inspection are consistent. Considering there may be cross-regional pollutant flows and governance spillover effects, it suggests that different stages of the CEPI have different impacts on air quality (21, 22).

Epidemiological evidence shows that older people, who make up a sizable and increasing segment of the global population, have long suffered from health problems caused by environmental factors. For instance, the familiar airborne particulate matter PM2.5 is a significant health hazard for people suffering from asthma, pneumonia, and respiratory diseases (23). However, declining lung function is a natural progression of aging, scientific evidence indicates that older people are much more vulnerable than other age groups when exposed to equivalent levels of air pollutants (24, 25). In addition, pre-existing diseases can be exacerbated due to unfavorable living conditions. To be specific, chronic diseases are more prevalent in older persons, and evidence proves that environmental contaminants increase the likelihood that chronic lung, heart, or circulatory problems may worsen (26, 27). Studies have demonstrated that particulate matter and all polluted gases in the air contribute to a higher risk of hospitalization and potentially exacerbate cardiac risks in the older adult (28). For example, sulfur dioxide (SO2) is more detrimental to susceptible people such as the older adult and people with lung diseases, causing illnesses such as bronchitis and bronchospasm (29). Studies in major hospitals in Hong Kong have shown that there is a correlation between the risk of emergency admissions for respiratory diseases and outdoor pollution. Between 1994 and 1995, admission to hospitals for all respiratory and chronic obstructive pulmonary diseases was highly correlated with sulfur dioxide concentrations, with 65-year-old people at higher risk (30). In addition, in terms of water pollution, poorly treated industrial wastewater discharges can contaminate groundwater and soil, posing a grave risk to human health (5). Liu found that water pollution can significantly increase older adult people’s levels of depression while severely diminishing their self-care and overall health, based on data from 20,435 residents over the age of 45 in 28 provinces in China. Harmful substances in water pose an extremely high health risk to older adults (31). Moreover, this health risk affects a sizable geographic area, and as water contaminants gradually enter the watershed, they are bound to have a detrimental impact on a large area. Pollutants accumulated in water bodies will predispose the older adult population to a wide range of diseases, including type 2 diabetes, hyperuricemia, rheumatic diseases, vascular diseases, myocardial infarction, neurological damage, liver damage, and cancer. These diseases, in turn, accelerate the aging process and further reduce the resistance of the older adult population, creating a vicious cycle (32). Research by Prüss and Bonjour suggested that between 13 and 37% of the global burden of disease could be prevented through environmental improvements, reducing the number of deaths by approximately 130,000 per year (33). Whereas the world’s population is rapidly aging, further improvements in the environmental factors associated with the level of health of the older adult are essential from both a social and scientific perspective (34).

Through a series of research on previous studies, this study proposes that there is currently a gap in research on the health impacts of the CEPI system on specific populations. Most of the existing studies directly analyze the economic, social, and environmental effects of the CEPI, focusing on sustainability, socialization costs, and the administrative game between the central and local governments (13–18, 20), but they fail to establish a direct analytical framework that focuses on the health status of the target populations. This has undoubtedly led to an underestimation of the derived social benefits of the CEPI and an underestimation of the social benefits of implementing the policy, which in turn biases the accuracy of the policy assessment. The study will focus on complementing this research gap, as well as contributing to a more comprehensive policy assessment of the CEPI. Furthermore, there is room for further improvement in the study of the transmission mechanisms between environmental regulatory policies and the health status of the older adult population. The existing literature also fails to answer the question of which pollutant emissions have the greatest impact on the health status of the older adult population, which will lead to a lack of focus on the future CEPI system and will not be conducive to the development of the system. This study addresses these issues in depth.

## 3 Empirical research design

### 3.1 Empirical models

As the CEPI is piloted in batches and implemented gradually, it can be modeled as a quasi-natural experiment. This study employs a difference-in-variances model to identify the influence of CEPI on the health of the older adult by applying the variations in the pilot policy at both the time and regional levels. The empirical models are constructed as follows:


(1) $$\mathrm{Shealt}h_{\mathrm{it}}=\alpha_{0}+\alpha_{1}DID_{\mathrm{it}}+\alpha_{2}\mathrm{Contro}l_{\mathrm{it}}+\gamma_{i}+\mu_{t}+\varepsilon_{\mathrm{it}}$$

In terms of intervention effectiveness evaluation, the DID model effectively combines “pre- and post-differences” and “whether there are differences,” to some extent controlling the influence of certain factors other than intervention factors. At the same time, adding other covariates that may affect outcome variables to the model further controlled for certain “suspected” influencing factors in the intervention group and control group, to supplement the defect that “natural experiments” cannot be completely random in sample allocation (35). Therefore, this article uses the DID model in the empirical part to explore CEPI’s impact on the health of the older adult.

In this model, *Shealth* is used to measure the health status of the older adult, with subscript i denoting the older adult, and t denoting the year. *DID* is a measure of CEPI. If the location where the older adult live piloted the CEPI system in year t, *DID* is assigned the value of 1; otherwise, it is 0, and this variable is equivalent to the interaction term in the traditional double-difference method. The selection of control variables is grounded on the study by Wang et al. (36) which identified their value in comparable contexts. *Control* represents the set of control variables, including a series of individual characteristics of the older adult and regional macro-socioeconomic variables. Individual characteristic variables of the older adult include age (*age*), gender (*gender*), marital status (*married*), whether living with their children (*hc*oresd), whether they registered in an urban area (*hukou*), whether they smoke (*smoke*), and whether they drink (*drink*). On this basis, macroeconomic and social variables are added, including the number of medical beds per thousand people in the region (*mbed*), the number of healthcare workers per thousand people (*mmpop*), disposable income *per capita* (*mincome*), economic growth rate (*rgdp*), and the share of the secondary industry (*cyjp*). This study controls for the regional fixed effects 𝛾_i_ and year fixed effects 𝜇_t_. The coefficient 𝛼_1_ measures the effect of the implementation of the CEPI, reflecting the net effect of the impact of the CEPI on the health of the older adult.

### 3.2 Construction of indicators

1. Health level of the older adult

This study draws on the methodology used in the study by Pang et al. (37), the self-assessed health score (*Shealth*), as a health metric to explore the relationship between medical insurance and health, and it generated desirable findings. Another reason for choosing *Shealth* as the explanatory variable is that it serves as a comprehensive and valuable judgment of an individual’s current level of health. Based on the results of individual choices in the survey question “What do you think of your health level?,” we assign a value of 1 to those who choose “very bad,” a value of 2 to those who choose “bad,” a value of 3 to those who choose “fair,” a value of 1 to those who choose “good,” and a value of 5 to those who choose “very good,” which is an ordered variable.

1. The central environmental protection inspection

**Figure 1:**
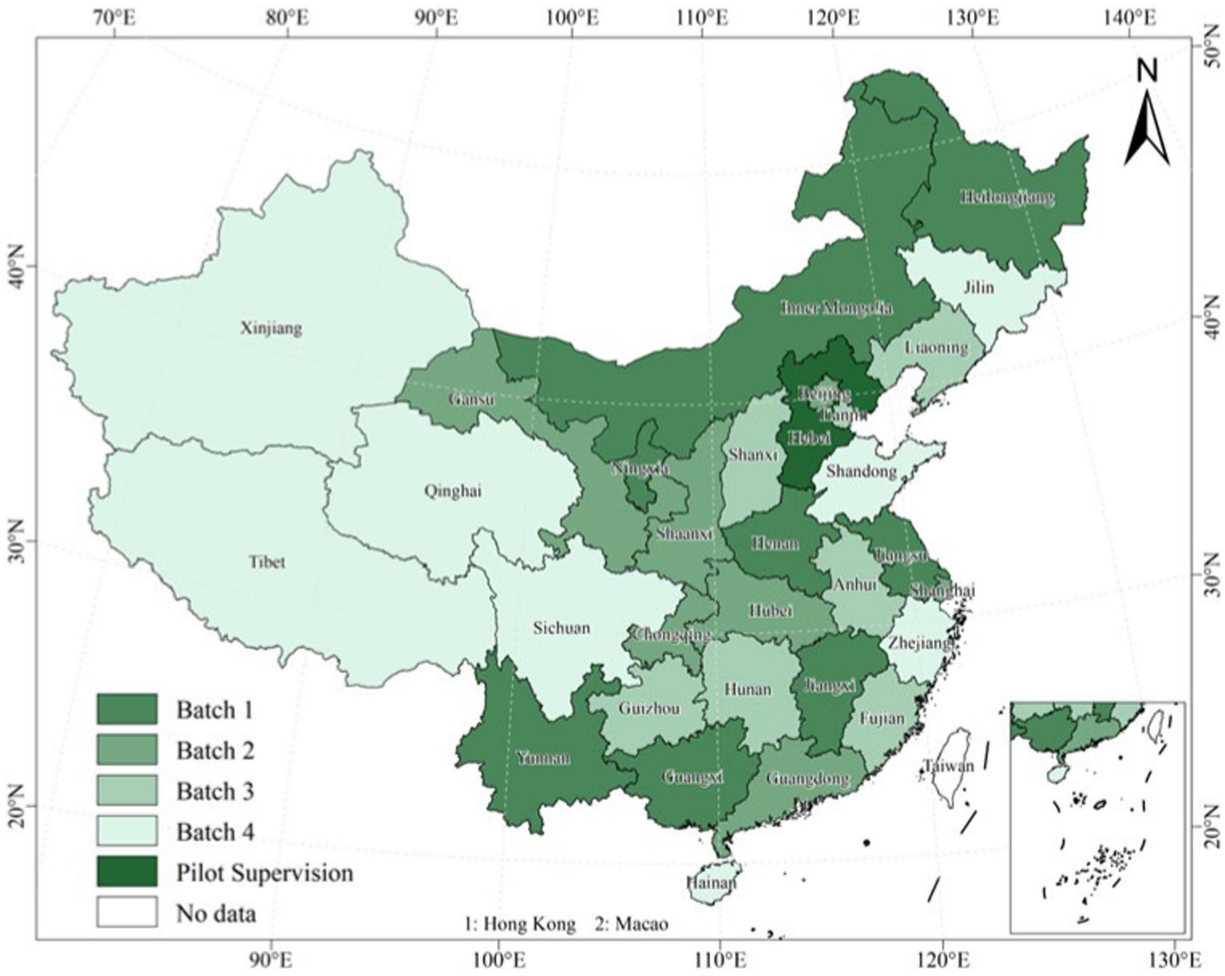
Pilot progress of the CEPI in China. Map Audit Number: GS(2020)4619 produced by the Ministry of Natural Resources of the People’s Republic of China.

The core explanatory variable is the dummy variable of CEPI. Referring to existing research, *DID* is assigned a value of 1 if the location the older adult live in piloted the CEPI system in year t, and 0 otherwise (17). Figure 1 depicts the CEPI pilot’s batch-by-batch progress.

### 3.3 Data description

The data come from the CHARLS database, and the regional socioeconomic variables come from the China Statistical Yearbook. The descriptive statistics of the data are shown in Table 1. It is a large-scale interdisciplinary survey project hosted by the National Development Research Institute of Peking University and executed by the China Social Science Research Center. This initiative combines international aging survey methodologies with the specific requirements of China’s aging population, as well as the needs of public health, socioeconomic fields, and other multidisciplinary areas when designing interviews and determining indicators. CHARLS employs a stringent multi-stage random sampling process. Initially, 150 districts and counties are chosen using the probability proportional to size (PPS) method, taking into account factors such as region, urban–rural classification, and *per capita* income. Subsequently, three village-level units, totaling 450 units, are selected from each district or county. CHARLS-GIS facilitates the mapping and labeling of living units within buildings to establish a sampling frame for households. From each household, an individual aged 45 or older is randomly chosen as the primary respondent, and their spouse is automatically included in the sample. This approach is widely recognized as the predominant sampling methodology in developing countries. CHARLS has conducted baseline surveys across 28 provinces, autonomous regions, and municipalities directly under central government administration since 2011. Follow-up surveys were completed in 2013, 2015, and 2018, with subsequent follow-ups planned at 1- to 2-year intervals. By the end of the 2018 national tracking survey, the sample encompassed 19,000 respondents from 12,400 households. Given the robustness of the research, this study combines data from CHARLS for the years 2013, 2015, and 2018, aligned with the research objectives. In 2013, 10,629 households and 18,264 respondents were included with an 88.3% tracking rate; in 2015, there were 11,797 households and 20,284 respondents with an 85.8% response rate; and in 2018, there were 10,524 households and 17,970 respondents with an 86.46% response rate (Refer to China Health and Aging Report (pku.edu.cn), P14). Since the CHARLS database started its survey in 2011 and the latest year is 2018, this study sets the sample period as 2011–2018. At the same time, samples with serious missing values are deleted, and continuous variables are indented by 1% up and down.

**Table 1:** Descriptive statistics for each of the main variables.

Variable	Obs	Mean	Std. Dev.	Min	Max.
Shealth	15,425	3.067	0.976	1	5
did	15,425	0.221	0.415	0	1
age	15,425	68.376	6.522	60	105
gender	15,425	0.461	0.498	0	1
married	15,425	0.214	0.41	0	1
hcoresd	15,425	0.384	0.486	0	1
hukou	15,425	0.736	0.441	0	1
Drink	15,425	0.298	0.458	0	1
Smoke	15,425	0.246	0.431	0	1
mbed	15,425	5.394	0.932	3.55	7.4
mmpop	15,425	5.989	1.323	3.31	15.46
mincome	15,425	22096.322	7761.994	9,739	64,183
rgdp	15,425	0.082	0.02	0.028	0.137
cyjg	15,425	0.429	0.057	0.165	0.62

## 4 Empirical results and analysis

### 4.1 Benchmark regression results

This study examines the impact of CEPI on the health of the older adult. The regression results of the model are presented in Table 2. In Table 2, column (1) gives the estimation results without adding any control variables, without controlling for year and region fixed effects, and the coefficient of *DID* is significantly positive at the 5% level, indicating that CEPI improves the health of the older adult. In column (2), after controlling for area and year fixed effects, the coefficient of *DID* remained significantly positive at the 5% level. In column (3), a series of individual characteristics variables of the older adult are added, including age (*age*), gender (*gender*), marital status (*married*), whether living with their children (*hcoresd*), registered permanent residence (*hukou*), whether they smoke (*smoke*), and whether they drink (*drink*). The regression results show that while the *DID* coefficient is still positive and significant at the 1% level, the goodness of fit of the model increased, suggesting that individual characteristic variables could potentially interpret the health of older adults. On this basis, macroeconomic and social variables such as the number of medical beds per thousand people in the region (*mbed*), the number of healthcare workers per thousand people (*mmpop*), disposable income *per capita* (*mincome*), economic growth rate (*rgdp*), and the share of the secondary industry (*cyjp*) are added. Column (4) displays the regression coefficients, where the coefficient of the core variable *DID* is significantly positive at the 10% level, which further confirms the robustness of the benchmark regression results.

**Table 2:** Benchmark regression results.

	(1)	(2)	(3)	(4)
	Shealth	Shealth	Shealth	Shealth
did	0.081**	0.095**	0.125***	0.09*
	(0.036)	(0.046)	(0.046)	(0.048)
age			0.006***	0.007***
			(0.002)	(0.002)
gender			−0.033	−0.035
			(0.037)	(0.037)
married			0.005	−0.004
			(0.04)	(0.04)
hcoresd			0.025	0.003
			(0.032)	(0.032)
hukou			0.309***	0.286***
			(0.036)	(0.037)
drink			−0.447***	−0.454***
			(0.037)	(0.037)
smoke			−0.084**	−0.081**
			(0.04)	(0.04)
mbed				0.016
				(0.033)
mmpop				−0.055**
				(0.024)
mincome				0***
				(0)
rgdp				3.059**
				(1.407)
cyjg				−0.682*
				(0.381)
Observations	15,425	15,425	15,425	15,425
*R*-squared	0.001	0.003	0.054	0.061

Standard errors are in parentheses.

***p < 0.01, **p < 0.05, and *p < 0.1.

***, **, and * denote significance at the 1, 5, and 10% levels, respectively, and the numbers in parentheses are robust standard errors clustered by firms.

### 4.2 Heterogeneity tests

1. Yangtze River basin and non-Yangtze River basin

Water pollution is a serious threat to human health (38). In areas with abundant water sources, toxic trace metals, coliforms, and other organic and inorganic pollutants can directly affect the health of local residents once surface and underground water sources are contaminated (39). CEPI might have a more prominent health impact on residents living in the Yangtze River Basin, which is rich in water resources. Columns (1) and (2) of Table 3 show the empirical results of regressing the samples from different regions separately. Column (1) shows the test results of cities in the Yangtze River Basin, with a coefficient of 0.915, which is significant at the 1% confidence level, while column (2) shows the coefficient for cities in the non-Yangtze River Basin, with a coefficient of 0.077, which is insignificant at the 10% confidence level, indicating that the CEPI has a greater impact on the health of the older adult in the Yangtze River Basin.

1. Urban and rural

**Table 3:** Heterogeneity between the Yangtze and non-Yangtze River Basin cities.

	Yangtze River basin cities	Non-Yangtze River basin cities
	Shealth	Shealth
did	0.915***	0.077
	(0.265)	(0.048)
age	−0.004	0.008***
	(0.01)	(0.003)
gender	0.063	−0.038
	(0.165)	(0.038)
married	−0.195	0.015
	(0.159)	(0.041)
hcoresd	−0.024	−0.004
	(0.128)	(0.033)
hukou	0.07	0.353***
	(0.145)	(0.036)
drink	−0.164	−0.474***
	(0.153)	(0.038)
smoke	−0.193	−0.074*
	(0.172)	(0.042)
chronic	−0.259*	0.021
	(0.15)	(0.03)
mbed	0.208*	−0.042*
	(0.117)	(0.023)
mmpop	0***	0***
	(0)	(0)
mincome	9.553	3.741***
	(6.586)	(1.309)
rgdp	−4.786	−1.008***
	(2.961)	(0.319)
cyjg	0.915***	0.077
	(0.265)	(0.048)
Observations	975	14,450
*R*-squared	0.06	0.063

Standard errors are in parentheses.

***p < 0.01, **p < 0.05, and *p < 0.1.

Existing research shows that compared with rural areas, urban areas have a higher degree of industrial agglomeration; thus, more serious urban pollution (40) and the impact of pollution on the health of urban residents is more significant (41). Therefore, there is possible heterogeneity in the impact of CEPI on rural and urban residents. Columns (1) and (2) in Table 4 demonstrate the empirical results of regressing the samples of rural and urban older adult people, respectively. Column (1) shows the regression results of rural older adult people, whose coefficient is insignificant at 10% confidence level, while column (2) shows the regression results of urban older adult people, with a coefficient of 0.152, which is significant at 1% confidence level, suggesting that the impacts of CEPI on the health of urban older adult people are more prominent.

1. With and without chronic diseases

**Table 4:** Heterogeneity between rural and urban.

	Rural	Urban
	Shealth	Shealth
did	−0.042	0.152***
	(0.091)	(0.055)
age	0.001	0.006**
	(0.005)	(0.003)
gender	0.085	−0.033
	(0.074)	(0.044)
married	−0.085	0.031
	(0.084)	(0.046)
hcoresd	−0.011	0.031
	(0.066)	(0.037)
drink	−0.406***	−0.415***
	(0.072)	(0.043)
smoke	0.097	0.011
	(0.082)	(0.047)
chronic	1.232***	1.142***
	(0.052)	(0.031)
mbed	−0.05	−0.007
	(0.056)	(0.034)
mmpop	−0.011	−0.085***
	(0.033)	(0.032)
mincome	0	0***
	(0)	(0)
rgdp	10.434***	2.204
	(2.381)	(1.54)
cyjg	0.153	−0.982**
	(0.567)	(0.421)
Observations	4,066	11,359
*R*-squared	0.067	0.059

Standard errors are in parentheses.

***p < 0.01, **p < 0.05, and *p < 0.1.

Air pollution not only increases the incidence of chronic diseases in residents (42) but also increases the health risk of patients with chronic diseases (43). Therefore, the study believes that there may be heterogeneity in the impact of CEPI on the older adult population with and without chronic diseases. The study utilized data concerning public chronic diseases from the CHARLS 2011 National Baseline Survey, serving as the initial point for a nationwide survey. In CHARLS, chronic diseases encompass long-lasting and persistent health conditions based on a medical diagnosis which include hypertension, dyslipidemia, diabetes, cancer, chronic lung diseases, liver diseases, heart problems, stroke, kidney disease, digestive issues, emotional or psychiatric problems, memory-related diseases, arthritis, and asthma, highlighting individual health conditions in the medical and public domain. Columns (1) and (2) in Table 4 show the empirical results of regression of the samples of older adult without and with chronic diseases, respectively. Column (1) shows the regression results of the older adult without chronic diseases, with a coefficient of 0.341, which is significant at the 1% confidence level, while column (2) in Table 5 shows the regression results of the older adult with chronic diseases, with a coefficient that remains insignificant at the 10% confidence level, which indicates that the effect of the CEPI on the health of the older adult without chronic diseases is more prominent (Table 5).

**Table 5:** Heterogeneity between with and without chronic diseases.

	Without chronic diseases	With chronic diseases
	Shealth	Shealth
did	0.341***	0.048
	(0.13)	(0.051)
age	0.01*	0.005*
	(0.006)	(0.003)
gender	−0.147	0.017
	(0.095)	(0.041)
married	−0.022	0.025
	(0.102)	(0.044)
hcoresd	−0.105	0.015
	(0.079)	(0.035)
drink	−0.272***	−0.461***
	(0.087)	(0.041)
smoke	0.006	−0.025
	(0.095)	(0.045)
hukou	0.342***	0.378***
	(0.092)	(0.038)
mbed	0.012	−0.024
	(0.081)	(0.036)
mmpop	−0.098	−0.038
	(0.063)	(0.026)
mincome	0*	0***
	(0)	(0)
rgdp	11.124***	0.734
	(3.622)	(1.533)
cyjg	−0.214	−0.431
	(0.927)	(0.419)
Observations	2,457	12,968
*R*-squared	0.026	0.014

Standard errors are in parentheses.

***p < 0.01, **p < 0.05, and *p < 0.1.

### 4.3 Robustness tests

To test the robustness of the previous empirical results, the study performs the following robustness tests.

1. Parallel trend test

The study draws on Ferrara et al. (44) to test the validity of the assumption through a “counterfactual” approach which varies the timing of policy implementation. Specifically, we have coordinated the CEPI timetable across all regions to be advanced by 1–3 years, and if the core variable *DID* is no longer significant, it reveals that the experimental group and the control group satisfy the common trend before the implementation of the inspections. In columns (1), (2), and (3) of Table 6, we report the results of the “counterfactual” regression, and it reflects that the core explanatory variable *DID* is not significant in the 1–3 years before the implementation of the CEPI, which implies that the parallel trend test has been passed.

1. Replacement of explanatory variables

**Table 6:** Parallel trend test.

	(1)	(2)	(3
	Shealth	Shealth	Shealth
didpre1	0.027		
	(0.039)		
didpre2		0.052	
		(0.034)	
didpre3			0.038
			(0.034)
age	0.007***	0.007***	0.007***
	(0.002)	(0.002)	(0.002)
gender	−0.035	−0.036	−0.036
	(0.037)	(0.037)	(0.037)
married	0.004	0.005	0.005
	(0.04)	(0.04)	(0.04)
hcoresd	−0.01	−0.012	−0.011
	(0.032)	(0.032)	(0.032)
hukou	0.321***	0.325***	0.324***
	(0.035)	(0.035)	(0.035)
drink	−0.459***	−0.458***	−0.458***
	(0.037)	(0.037)	(0.037)
smoke	−0.076*	−0.077*	−0.077*
	(0.04)	(0.04)	(0.04)
chronic	0.007	0.016	0.013
	(0.033)	(0.033)	(0.034)
mbed	0***	0***	0***
	(0)	(0)	(0)
mmpop	2.866**	2.85**	2.82**
	(1.41)	(1.407)	(1.41)
mincome	−0.629*	−0.616	−0.596
	(0.381)	(0.381)	(0.382)
rgdp	0.007***	0.007***	0.007***
	(0.002)	(0.002)	(0.002)
cyjg	−0.035	−0.036	−0.036
	(0.037)	(0.037)	(0.037)
Observations	15,425	15,425	15,425
*R*-squared	0.063	0.063	0.063

Standard errors are in parentheses.

***p < 0.01, **p < 0.05, and *p < 0.1.

The degree of incapacity of the older adult is selected as a substitution variable for the health of the older adult, and the higher the degree of incapacity, the lower the health of the older adult. Columns (1) to (4) are the benchmark regression analysis, regressions controlling for region and time-fixed effects, regressions controlling for individual characteristics variables, and regressions controlling for individual characteristics as well as regional macro-variables. The coefficients of the four regression results are all negatively significant at the 10% level, suggesting that CEPI improves the situation of older adult incapacitation, which increases the credibility of basic empirical conclusions in this study.

1. Placebo test for exclusion of randomized outcomes

**Figure 2:**
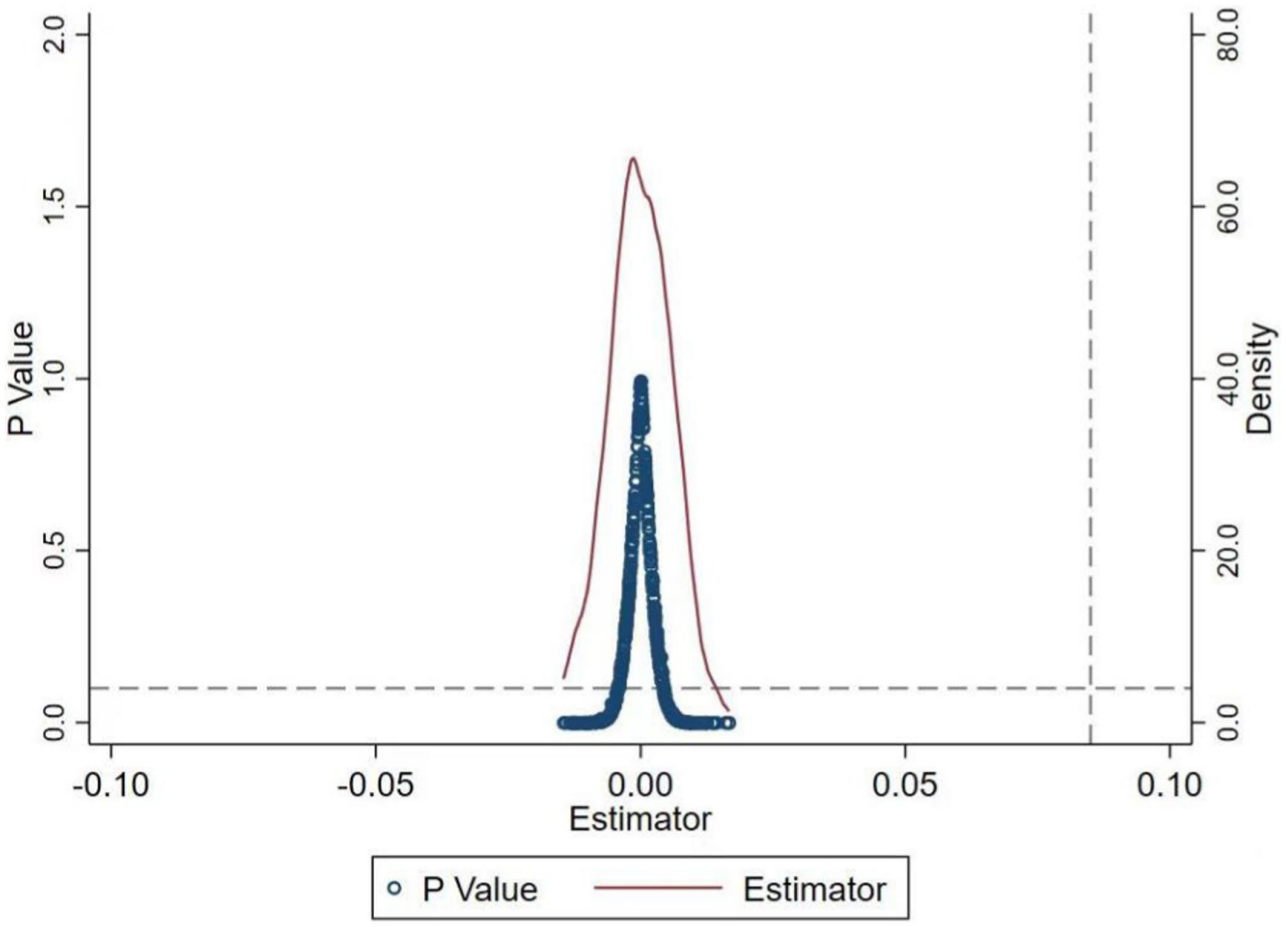
Placebo test chart.

To rule out the effect of randomized results, drawing on Chetty et al. (45)'s approach, the year and region in which CEPI was implemented were randomized, and this process was repeated 1,000 times for the placebo test. The results, as shown in Figure 2, manifest that the randomized simulation yields a distribution of regression coefficients of approximately 0, while the coefficients of the benchmark regression are completely independent of this coefficient distribution. This represents that the empirical results observed in Table 7 in this study are not due to randomness or chance factors.

**Table 7:** Replacement of explanatory variables.

	(1)	(2)	(3)	(4)
	adl	adl	adl	adl
did	−0.119***	−0.135**	−0.053*	−0.121**
	(0.045)	(0.057)	(0.028)	(0.06)
age			0.024***	0.048***
			(0.001)	(0.003)
gender			−0.076***	−0.308***
			(0.022)	(0.047)
married			0.089***	0.159***
			(0.024)	(0.047)
hcoresd			0.052***	0.079**
			(0.019)	(0.039)
hukou			−0.115***	0.396***
			(0.022)	(0.046)
drink			−0.052**	−0.22***
			(0.024)	(0.047)
smoke			0.191***	−0.089*
			(0.021)	(0.052)
mbed				0.068
				(0.042)
mmpop				−0.158***
				(0.035)
mincome				0***
				(0)
rgdp				−8.635***
				(1.721)
cyjg				−0.152
				(0.506)
Observations	15,425	15,425	15,425	15,425
*R*-squared	0.001	0.004	0.024	0.029

Standard errors are in parentheses.

***p < 0.01, **p < 0.05, *p < 0.1.

#### 4.3.1 PSM-DID

Propensity score-matched double differencing was used to verify the robustness of the regression results to rule out bias resulting from potentially unobservable factors. The study uses the control variables as the matching variables and regresses the samples obtained through methods including nearest neighbor matching, kernel matching, and radius matching (46). The results are shown in columns (1) to (3) of Table 8. The regression results show that the effect of the CEPI on the health of the older adult is still significantly negative at the 1% level, indicating that the benchmark regression results are robust.

**Table 8:** PSM-DID test results.

	(1)	(2)	(3)
	Nearest neighbor matching	Kernel matching	Radius matching
	Shealth	Shealth	Shealth
did	0.142***	0.082**	0.075*
	(0.053)	(0.04)	(0.04)
age	0.013***	0.013***	0.013***
	(0.003)	(0.002)	(0.002)
gender	−0.11***	−0.069**	−0.072**
	(0.04)	(0.03)	(0.03)
married	0.008	0.015	0.017
	(0.045)	(0.032)	(0.032)
hcoresd	−0.018	−0.019	−0.025
	(0.034)	(0.025)	(0.025)
hukou	0.329***	0.333***	0.331***
	(0.04)	(0.029)	(0.029)
drink	−0.472***	−0.447***	−0.442***
	(0.039)	(0.029)	(0.029)
smoke	−0.019	−0.103***	−0.101***
	(0.044)	(0.032)	(0.032)
mbed	0**	0***	0***
	(0)	(0)	(0)
mmpop	0.832	1.557	1.332
	(1.529)	(1.101)	(1.115)
mincome	−0.392	−0.764**	−0.624*
	(0.437)	(0.31)	(0.325)
rgdp	0.142***	0.082**	0.075*
	(0.053)	(0.04)	(0.04)
cyjg	0.013***	0.013***	0.013***
	(0.003)	(0.002)	(0.002)
Observations	8,253	15,294	15,282
*R*-squared	0.063	0.062	0.062

Standard errors are in parentheses.

***p < 0.01, **p < 0.05, and *p < 0.1.

### 4.4 Mechanism tests

**Figure 3:**
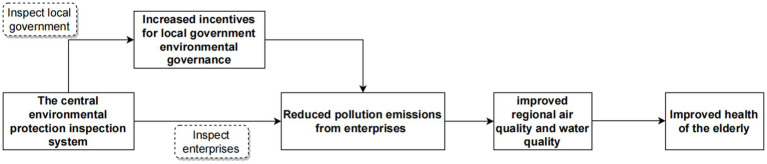
Mechanism of the impact of CEPI on the health of the older adult.

Combined with the results of the benchmark regression, CEPI can improve the health of the older adult. The study will continue to conduct mechanism testing referring to theoretical analysis. The Central Environmental Protection Inspectorate oversees both the government and the enterprise simultaneously, not only regulating the emission behavior of enterprises as part of ordinary environmental regulation but also supervising whether the government enforces the environmental laws and regulations properly. In China’s institutional system of selecting and appointing leading cadres, local officials are appointed by the Party Central Committee in a hierarchical manner, forming a tiered system of delegation and representation with the central government (47). Among them, the economic growth of the jurisdiction is an important criterion for the selection of cadres, which incentivizes local officials to prioritize economic growth at the expense of environmental protection and the wellbeing of residents (48). Furthermore, the local environmental protection bureau in the city is financially and staff-wise accountable to the corresponding level of local government (49). This implies that local governments play dual roles as both “players” and “referees,” often overlooking environmental pollution to secure their political future. Consequently, environmental laws and regulations are inadequately enforced, jeopardizing the wellbeing of residents. The deployment of the Central Environmental Protection Inspection (CEPI) has brought about a shift in this scenario. During the deployment period, if a pollution incident is reported in a district, local and municipal officials can be held responsible by the inspection group. This not only impacts the career prospects of local officials but may also result in more severe penalties, such as demotion (13). The inaugural round of CEPI led to the accountability of 18,000 individuals and the administrative and criminal detention of 2,264 people (11). Therefore, CEPI increases the accountability of local governments: The strong intervention of the central authority brings strong political pressure and deterrence to local officials, which will significantly increase local governments’ attention to environmental issues (18) and then increase pollution control. In light of these developments, the CEPI and local government environmental governance incentives will lead to the stringent restriction and regulation of the production and emissions of pollution-intensive firms with high energy consumption and pollution. CEPI not only scrutinizes the local governments’ adherence to environmental protection laws but also possesses the authority to directly impose penalties and, in extreme cases, shut down illicit enterprises. The inaugural round of CEPI significantly contributed to the resolution of over 150,000 ecological and environmental issues affecting residents and initiated legal actions to penalize more than 40,000 enterprises. This will reduce the degree of pollution emissions in the inspected areas (50), thereby improving regional air and water quality. Numerous research studies have shown that it will in turn benefit the health of the older adult (24, 25). Building on these analyses, this study argues that CEPI improves regional environmental quality through a range of transmission mechanisms, which in turn contributes to improving the health of the older adult. We take the local governments’ environmental governance motivation, the degree of regional pollution emission, and the Air Quality Index (AQI) as mediating variables to examine the mechanism of CEPI’s impact on the health of the older adult.^2^ The possible mechanism of impact is shown in Figure 3.

1. The mediating mechanism test of local governments’ environmental governance motivation

**Figure 4:**
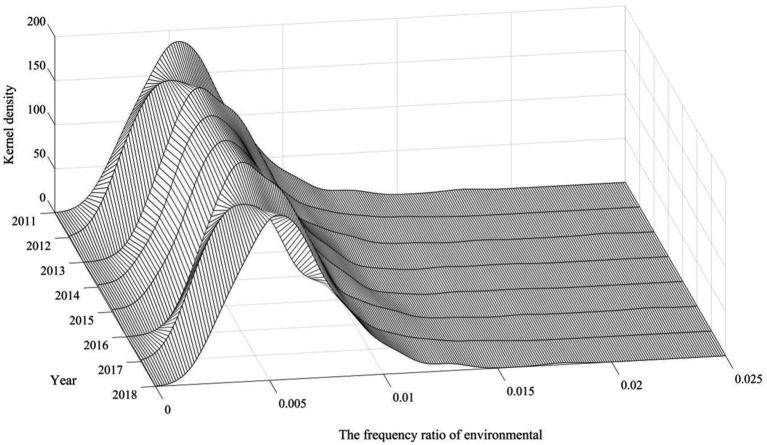
Kernel density profile of local government’s environmental governance motivation.

Drawing on the study of Chen and Chen (51), the study proposes the following specific steps for constructing government environmental governance indicators: first, manually collect government work reports of 287 cities from years 2011 to 2018; second, process the text of the government work reports by word division; finally, count the frequency of the occurrence of environment-related words and calculate their proportion to the total number of word frequencies of the full text of the government reports. The environment-related words include environmental protection, pollution, energy consumption, emission reduction, sewage disposal, ecology, green, low carbon, air, chemical oxygen demand, sulfur dioxide, carbon dioxide, PM10, and PM2.5. The ratio of the total number of the above words to the total number of words in the text was used as a substitution variable for the governments’ environmental governance motivation. Figure 4 plots the kernel density profile of the proportion of environment-related vocabulary in the government work report. The proportion of environment-related vocabulary in government work reports has been increasing from 2011 to 2018, which is very much in line with the government’s increasing attention to environmental issues (51). On this basis, this study explores the impact of CEPI on local governments’ environmental governance motivation, and the regression results are shown in Table 9. It is found that the CEPI improves local governments’ environmental governance motivation.

1. The mediating mechanism test of the degree of regional pollution emission and the AQI

**Table 9:** Regression results of the impact of the CEPI on local governments’ environmental governance motivation.

	(1)
	Local governments’ governance motivation
did	0.001***
	(0)
rgdp	0.022***
	(0.002)
cyjg	0.008***
	(0)
_cons	0
	(0)
Observations	1,008
*R*-squared	0.185

Standard errors are in parentheses.

***p < 0.01, **p < 0.05, and *p < 0.1.

Given the availability of regional and municipal emissions data, the study selects industrial wastewater emissions, industrial sulfur dioxide emissions, and industrial soot emissions in each prefecture-level city as mediating variables to explore the impact of the CEPI on the above variables. The regression results, shown in Table 10, indicate that the CEPI significantly reduces industrial wastewater emissions, industrial sulfur dioxide emissions, and industrial soot emissions, which reveal that the intensity of regional emissions may be a possible pathway through which CEPI influences the health of the older adult. In addition, we explored the impact of CEPI on the Air Quality Index (AQI) based on previous studies (52). The AQI covers pollutants such as PM10, SO2, NO2, PM2.5, CO, and O3, with the higher the AQI value, the worse the air quality. The regression results are shown in the fourth column of Table 10, and the coefficients are significantly negative at the 1% level, indicating that the central environmental protection inspection system improves air quality.

**Table 10:** Regression results of the impact of the CEPI on regional emissions.

	(1)	(2)	(3)	(4)
	Industrial wastewater emissions	Industrial sulfur dioxide emissions	Industrial soot emissions	AQI
did	−1602.573***	−49.34	−10.336***	−9.262***
	(247.546)	(1762.877)	(0.634)	(1.721)
rgdp	69011.619***	48547.095	512.334***	−132.803***
	(6543.342)	(46752.432)	(16.488)	(35.538)
cyjg	16790.348***	57385.979***	15.931***	128.391***
	(2090.92)	(14915.944)	(5.296)	(9.985)
_cons	−12277.092***	39824.73***	−18.039***	31.65***
	(1191.856)	(8551.376)	(3.028)	(6.674)
Observations	1,008	1,008	1,008	1,008
*R*-squared	0.115	0.16	0.195	0.372

## 5 Conclusion and policy implications

### 5.1 Conclusion

(1) The Central Environmental Protection Inspectorate improves the overall health status of the older adult as the study results remain significant through the parallel trend test, PSM test, substitution variable test, and placebo test. (2) The heterogeneity analysis demonstrates that CEPI has a more significant effect on the health of the older adult in the Yangtze River Basin, the older adult residing in urban areas, and older adult populations free of chronic diseases. (3) In terms of the mechanism of influence, the CEPI system activates local governments’ motivation for environmental governance and reduces industrial wastewater emissions, industrial sulfur dioxide emissions, and industrial soot emissions. It has improved air quality and created a good living environment for the older adult.

### 5.2 Policy implications

1. The government should strengthen the long-term environmental protection supervision mechanism and perfect the public engagement system in environmental supervision. It should also set emission standards that are consistent with regional realities and close down enterprises with high levels of pollutant emissions, to prevent them from generating more pollutant emissions and harming society. Accordingly, to increase the economies of scale of environmental regulation for the improvement of older adult health, it is also necessary to improve local governments’ and relevant departments’ environmental governance capabilities as environmental regulation is intensified.
2. There should be a cross-regional and cross-sectoral mechanism for environmental regulation in the Yangtze River basin. The extensive influence of the CEPI system on the health of older adult populations in the cities of the Yangtze River Basin reflects the value of environmental governance in the region. The government should establish a well-functioning system of pollution monitoring and early warning in the Yangtze River basin to keep abreast of environmental pollution and to prevent threats to older adult populations. Cooperation and support from different governmental sectors are essential as well, and the government should establish a cross-sectoral cooperation mechanism that involves relevant departments such as environmental protection, health, planning, and social security, to form a synergy to promote pollution management and the improvement of the health of the older adult in the Yangtze River Basin.
3. Constructing an age-friendly city should be a priority. Because older adult generations in cities typically deal with more intense and complicated environmental pollution issues, environmental governance has a significant influence on their health. For this reason, on the one hand, the government should formulate comprehensive urban environmental protection policies, including air quality improvement, water pollution prevention, and control. These policies should prioritize the older adult’s health and ensure that they live in a relatively clean and quiet environment. On the other hand, creating age-friendly cities means improving the design of public spaces, providing leisure and fitness facilities, and optimizing community medical services, which are beneficial for the quality of life and wellbeing of the older adult.

### 5.3 Limitations

First, despite the comprehensive health and life questionnaire provided by CHARLS, it may not encompass all potential health-influencing factors, possibly leading to omitted variable issues. Second, our study primarily focused on elucidating how CEPI influences the older adult’s health, but it did not delve into complex mediating effects, such as moderation and multiple chained mediation. Further research could consider employing methods such as structural equation modeling to more thoroughly explore the relationships and magnitudes of these mediating variables. Finally, due to data availability, the empirical part of the mechanism test in this study only deals with the AQI and does not test the effect of CPCI on water quality; in the future, we will collect as much water quality data as possible to improve the mechanism test.

## Data availability statement

The original contributions presented in the study are included in the article/supplementary material, further inquiries can be directed to the corresponding author.

## Ethics statement

Ethical approval was not required for the study involving humans in accordance with the local legislation and institutional requirements. Written informed consent to participate in this study was not required from the participants or the participants’ legal guardians/next of kin in accordance with the national legislation and the institutional requirements.

## Author contributions

JY: Writing – original draft, Writing – review & editing, Conceptualization, Methodology. JD: Writing – original draft, Writing – review & editing. LY: Writing – original draft, Writing – review & editing. LL: Writing – original draft. XH: Funding acquisition, Project administration, Writing – review & editing, Supervision.

## Footnotes

^1^Local government in China refers to people’s governments at all levels relative to the Central People’s Government (State Council), and the local governments studied in this paper are mainly at the municipal level. In addition, the areas mentioned herein are also areas within the municipal boundaries.

^2^Due to data availability, the empirical part of the mechanism test in this paper only deals with the AQI and does not test the effect of CPCI on water quality.
